# In-Class AI Use and Attitudes Among University Students: The Different Mediating Roles of Cognitive Relief and Cognitive Offloading

**DOI:** 10.3390/bs16061014

**Published:** 2026-06-17

**Authors:** Wenqiang Fan, Lu Cheng, Yanxiao Wang, Qi Zhao, Yaodong Li

**Affiliations:** 1 School of Humanities & Social Sciences, Beihang University, Beijing 100191, China; 2 School of Electronic & Information Engineering, Beihang University, Beijing 100191, China; 23373460@buaa.edu.cn; 3 School of Software Engineering, Beihang University, Beijing 100191, China; yanxiaowang@buaa.edu.cn; 4 School of Artificial Intelligence, Beihang University, Beijing 100191, China; 24421031@buaa.edu.cn

**Keywords:** cognitive relief, cognitive offloading, competitive mediation, complementary mediation, generative artificial intelligence

## Abstract

AI use is associated with both cognitive relief and cognitive offloading, leading to uncertainty in how users make value judgments and decisions. This study focuses on in-class AI use and explores the perceptions of cognitive relief and cognitive offloading among university students, as well as the distinct mediating mechanisms through which these factors shape the attitudes of students. Based on questionnaire data from 287 respondents, structural equation modeling and bootstrap methods were employed to test the research hypotheses. The results show that cognitive relief exerts a complementary mediating effect between AI use and attitudes, whereas cognitive offloading functions as a competitive mediator. The two mechanisms produce opposing effects on students, with cognitive relief demonstrating a stronger overall mediating effect. These findings suggest that educators should guide students toward a more nuanced understanding of AI use to mitigate confusion and its potential negative psychological consequences. Moreover, educators and institutions should leverage AI to provide cognitive relief for higher-order learning activities, thereby enhancing the engagement, motivation, and deeper learning processes of students, while simultaneously implementing reflective and critical thinking practices to guard against the risks of cognitive offloading. This study is limited by its single-institution convenience sample and reliance on self-reported data; future research incorporating qualitative methods such as interviews and classroom observations is encouraged to further validate and extend these findings.

## 1. Introduction

The rapid rise in generative artificial intelligence (AI) is reshaping teaching and learning in higher education. Tools such as ChatGPT have rapidly permeated academic writing and problem-solving practices, emerging as increasingly popular learning aids among university students (Baek et al., 2024; Guo et al., 2025).

However, AI use exerts a dual impact on learning and cognition, reflecting a tension between cognitive relief and cognitive offloading (Chirayath et al., 2025; Shalu et al., 2025). As an external cognitive tool, AI can alleviate the cognitive loads of users; yet, excessive reliance on it may deteriorate cognitive abilities (Dergaa et al., 2024). Prior research similarly suggests that while AI can enhance learning efficiency and optimize personal learning processes, it may also weaken core cognitive skills such as critical thinking, problem solving, and long-term memory by excessively replacing active thinking (Jose et al., 2025a). Consistent with this duality, students often regard AI as both an indispensable tool for enhancing efficiency in writing, learning, and research and a potential threat to learning autonomy and deep knowledge construction (Khalil et al., 2025).

These tensions place users in a dilemma: on the one hand, avoiding AI may reduce efficiency, technological opportunities, and competitiveness; on the other hand, over-reliance may introduce risks such as cognitive deterioration and cognitive debt (Dergaa et al., 2024; Kosmyna et al., 2025; Miranda et al., 2025). Existing studies have recognized the dual nature of AI usage, yet they have paid little attention to how this duality shapes users’ attitudes and is reflected in their contradictory experiences. A key point of contention concerns whether AI use leads to cognitive relief or cognitive offloading. This study focuses on these two contrasting mechanisms to examine how they impact attitude formation.

The central tension driving this research lies in the inherently contradictory nature of AI use in higher education: the same tools that promise cognitive relief may simultaneously induce cognitive offloading. On the one hand, cognitive relief refers to the technical support and optimization of thinking processes provided by AI, enabling humans to handle complex tasks more efficiently and to allocate mental resources to creation and decision making (Becker et al., 2025; Haider et al., 2024; Shalu et al., 2025). On the other hand, cognitive offloading is associated with risks such as the deterioration of inherent cognitive abilities and weakened critical thinking arising from excessive reliance on external tools (Gerlich, 2025a, 2025b; Malik et al., 2023; Oakley et al., 2025). Rather than treating AI as either uniformly beneficial or detrimental, this study argues that the interplay between these two mechanisms (efficiency versus risk) forms the core paradox that shapes the attitudes of users toward AI in educational contexts.

Beyond the direct effects of AI use on cognitive processing, prior research has identified the following critical psychological constructs that shape how individuals interact with AI systems: (1) AI literacy: Higher levels of AI literacy have been associated with more realistic expectations of AI capabilities and reduced technostress (Rožman et al., 2025). (2) Trust in AI: Trust in AI has been shown to influence the willingness to delegate tasks to AI and to accept AI-generated recommendations (Gerlich, 2025a). (3) Digital self-efficacy: Improving the digital self-efficacy of learners helps enhance their interactive experience and serves as a key psychological mechanism linking digital literacy and AI interaction perception (Ren et al., 2025). (4) Academic stress: As a key external situational factor, academic stress fuels reliance on AI among college students and induces cognitive offloading and motivation decline via the use of AI as a coping strategy (Miranda et al., 2025). From a methodological standpoint, existing studies have been predominantly survey-based, and qualitative research remains underrepresented.

This study constructs a model of attitude formation toward in-class AI use among university students and examines their perceptions of cognitive relief and cognitive offloading, as well as the different mediating roles of these contrasting mechanisms in shaping their attitudes toward AI usage in classroom contexts. This study yields two key contributions. First, theoretically, this study advances understanding of AI use by empirically validating a dual-path model of attitude formation, thereby establishing the contradictory relationship between cognitive relief and cognitive offloading as a core driver of user attitudes. Second, empirically, it presents quantitative data documenting the simultaneous coexistence of cognitive relief and cognitive offloading in AI learning scenarios. This study integrates these two-dimensional variables in a unified analytical framework, addressing the limitation of prior research that has only examined the two mechanisms independently.

## 2. Hypotheses and Research Model

### 2.1. Research Model

**Figure 1:**
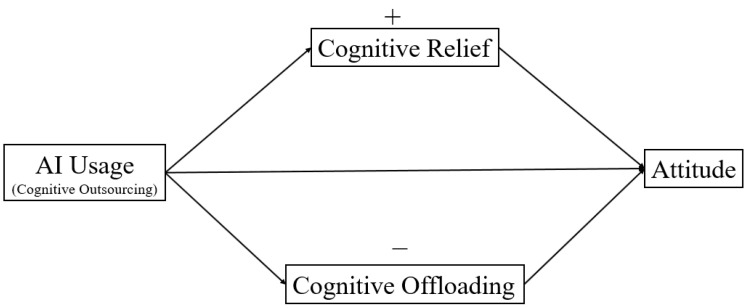
Research model diagram.

This study develops a model of attitude formation toward AI use based on existing theories (Figure 1). In the context of AI use, users may delegate part or all of their cognitive activities to external AI tools, a process referred to as cognitive outsourcing (used as a neutral term herein) (Zhang et al., 2025; Jose et al., 2025b).

Based on Cognitive Load Theory (Sweller, 1988), AI can significantly reduce extraneous cognitive load (e.g., information searching, format processing) by processing information efficiently and providing explanations and examples. This enables users to allocate greater cognitive resources to germane load associated with deeper thinking and schema construction and thereby providing cognitive relief (used as a positive term herein) (Chirayath et al., 2025; Khalil et al., 2025; Rafner et al., 2023; Wang et al., 2025).

Meanwhile, AI use may also lead to cognitive offloading (used as a negative term herein). When users entrust core cognitive tasks such as analysis, memory, and reasoning to AI, rely on it to generate answers, and replace it for their own cognitive processes, they may reduce their engagement in independent cognitive processing, a phenomenon referred to as cognitive offloading (Zhai et al., 2024).

Cognitive relief generally refers to the subjective, affective-perceptual sense of reduced psychological burden experienced by an individual—that is, the feeling of the alleviation of cognitive demand. In contrast, cognitive offloading refers to the behavioral process of delegating cognitive tasks to external tools or resources and is often conceptualized as an adaptive strategy for managing limited cognitive resources (Cavicchi et al., 2025). Although the two are related (cognitive offloading can lead to cognitive relief), they are not the same construct. Whether cognitive offloading is beneficial or detrimental depends on the specific usage context and task demands, as well as individual differences.

The conceptualization of cognitive offloading remains ambiguous, and the possibilities of it being viewed as either a negative or a positive phenomenon coexist. Risko and Gilbert (2016) define cognitive offloading as the replacement of internal cognitive processing with external physical operations to alleviate cognitive strain. They also note that cognitive offloading presents dual effects, encompassing both positive and negative facets. Cognitive offloading refers to transferring cognitive functions such as memory and computation to external tools, and its downsides have increasingly become a focal point of academic debate, as it has been re-examined particularly in the artificial intelligence era as a critical issue that is associated with cognitive degradation risks. Risko and Gilbert (2016) imply the negative dimension of cognitive offloading in their discussion: improved external efficiency is achieved at the cost of internal cognitive capabilities. This indicates that cognitive offloading is not an unconditional cognitive optimization strategy, and that its effectiveness hinges on the solid cognitive foundations of individuals, as reasonable cognitive offloading can only exert its instrumental value when individuals possess sound internal cognitive abilities. Grinschgl et al. (2021) found that a higher degree of cognitive offloading correlated with a faster and better performance in immediate tasks but led to the significantly poorer retention of offloaded information in subsequent memory tests. This empirical finding reveals a trade-off between short-term efficiency and long-term memory that is associated with cognitive offloading. Jose et al. (2025b) have further proposed the concept of disruptive offloading to describe the potential adverse impacts of cognitive offloading on memory, metacognition, attention, and learner autonomy. Given that a growing body of studies conceptualizes cognitive offloading as a risk-laden construct (e.g., Miranda et al., 2025; Gerlich, 2025b), in this study, we treat it as a negative construct and adopt items measuring the perceived negative consequences of AI use to operationalize it.

Cognitive relief and attitude are two theoretically distinct constructs. Cognitive relief refers to the perceived reduction in cognitive burden in AI-assisted learning, while attitude represents an individual’s evaluative judgment regarding the AI use. Specifically, cognitive relief addresses whether AI lessens the mental effort in learning activities, whereas attitude concerns whether students perceive AI use as beneficial, appropriate, or favorable. A student may experience cognitive relief (e.g., finding AI helpful for solving a problem) without necessarily holding a positive attitude toward AI use in general, and vice versa.

In this model, cognitive relief and cognitive offloading occur simultaneously during AI use. Cognitive outsourcing may lead to either outcome depending on an individual’s cognitive regulation. Accordingly, the attitudes of users toward AI use are shaped not by a single mechanism but by the dynamic interaction and trade-off between these two cognitive processes in specific usage scenarios.

Why cognitive relief leads to more favorable attitudes toward AI. Cognitive relief refers to the immediate, subjective reduction in perceived mental workload achieved through the use of an external tool—in this case, AI. When users experience cognitive relief, they associate AI with positive outcomes, such as reduced effort, time savings, and lower mental strain. Drawing on affect-as-information theory (Schwarz, 2012) and reinforcement learning principles, the positive affective experience accompanying cognitive relief serves as a reward signal. Consequently, users develop more favorable attitudes toward AI because it is repeatedly paired with a subjectively beneficial state (i.e., relief). In other words, cognitive relief acts as a positive reinforcer that shapes attitudes through experiential learning.

Why cognitive offloading leads to less favorable attitudes toward AI. In contrast, cognitive offloading may lead to less favorable attitudes toward AI through two interrelated mechanisms: First, the metacognitive awareness of under-engagement plays a key role. Users may experience a lack of personal cognitive investment when they engage in cognitive offloading via AI (e.g., accepting AI-generated answers without critical thinking) and, over time, may become aware that they are not fully understanding, retaining, or verifying information, triggering a sense of cognitive incompleteness or epistemic uncertainty. Second, attribution and negative attitude formation further reinforces this effect. According to self-perception theory (Bem, 1972) and cognitive dissonance theory (Festinger, 1957), if a user repeatedly observes themselves relying on AI in a way that bypasses their own reasoning, then they may infer that their own competence is being undermined, or that AI is unacceptably “taking over”, leading to a less favorable attitude toward AI—not because of AI failure but because the user’s own sense of agency and mastery is threatened.

Thus, the negative effect of cognitive offloading on attitudes is theoretically distinct from the positive effect of cognitive relief: the former involves the risk of self-perceived cognitive passivity, while the latter involves the reward of immediate ease.

We introduce a dual-process theory (Kahneman, 2011) to further clarify this distinction: cognitive relief operates through the fast, affective, experiential system (System 1), while cognitive offloading implicates the metacognitive monitoring of one’s own cognitive effort (System 2). According to this theoretical framing, we propose that the same AI application can generate both positive and negative attitude components, depending on whether the user focuses on the relief itself or on the loss of personal cognitive investment.

### 2.2. AI Usage and Cognitive Relief

According to the Cognitive Load Theory (Sweller, 1988) and Extended Mind Theory (Clark & Chalmers, 1998), AI, as an external cognitive tool, can transfer part of users’ cognitive tasks to external systems, thereby reducing their working memory demands and providing cognitive relief (Becker et al., 2025; Sana & Dissanayake, 2025; Shalu et al., 2025). AI is particularly effective at handling repetitive, structured, and low-level cognitive tasks (Dergaa et al., 2024; Haider et al., 2024), and the automated processing of AI reduces cognitive steps that would otherwise interfere with task performance, thereby reducing extraneous cognitive load (Chirayath et al., 2025; Jose et al., 2025a). AI also facilitates the comprehension of complex material and supports more efficient cognitive processing by bridging knowledge gaps and providing task scaffolding, thereby managing intrinsic cognitive load (Becker et al., 2025; Kung et al., 2023).

When AI undertakes low-order tasks and simplifies complex information, the freed-up cognitive resources of learners can be reallocated to higher-order thinking activities, such as in-depth analysis, critical thinking, creative synthesis, and strategic decision making, thereby supporting germane cognitive processing (Khalil et al., 2025; Rafner et al., 2023; Song, 2025; Wang et al., 2025).

In classroom settings, AI assists students in comprehending unfamiliar concepts, processing complex ideas, engaging in reflection on course content, and expanding their knowledge scope (Granström & Oppi, 2025; Guo et al., 2025; Xia et al., 2025). As a result, learning becomes more effortless.

Hence, this study proposes the following hypothesis:

**H1.**  *University students’ in-class AI use and perceptions of cognitive relief are positively correlated.*

### 2.3. AI Usage and Cognitive Offloading

Based on Cognitive Offloading Theory (Risko & Gilbert, 2016), AI usage allows individuals to offload cognitive processes to external tools, thereby reducing their mental effort and reliance on independent thinking (Kosmyna et al., 2025). In educational contexts, students may accept AI-generated content uncritically and forgo cognitive processes that would otherwise be required for independent task completion, thereby resulting in cognitive offloading (Gerlich, 2025b; Malik et al., 2023; Zhai et al., 2024).

AI tools often function as substitutes rather than as auxiliary tools, shifting the role of students from active knowledge constructors to more passive recipients. This shift offloads cognitive resources that would otherwise be devoted to the development of metacognition and self-regulation (Grassini, 2023; Kosmyna et al., 2025). Moreover, AI usage may lead students to favor heuristic approaches over deliberate step-by-step reasoning and analysis, thereby bypassing the time-consuming and effortful processes involved in constructing logical arguments (Oakley et al., 2025; Zhai et al., 2024). In many cases, users make only minor adjustments to AI-generated content, resulting in reduced creativity and shallow information processing (Alhur et al., 2025; Haider et al., 2024; Jose et al., 2025a). At the same time, students may not fully recognize declines in their own cognitive engagement (Dergaa et al., 2024).

In classroom settings, AI can quickly provide well-structured and fluently articulated answers, potentially shifting motivation away from active thinking toward efficiency gains (Gerlich, 2025a; Malik et al., 2023; Yatani et al., 2024) and attenuating the instincts of students to question and verify information (Haider et al., 2024; Khalil et al., 2025; Zhai et al., 2024). Hence, this study proposes the following hypothesis:

**H2.**  *University students’ in-class AI use and perceptions of cognitive offloading are positively correlated.*

### 2.4. The Mediation of Cognitive Relief and Cognitive Offloading Between AI Use and Attitudes

Attitudes influence behaviors, and individuals’ behavioral experiences, in turn, shape or reinforce their value tendencies toward such behaviors (Olson & Stone, 2005). In the context of AI as a new technology, students’ attitudes toward its use are still evolving, with early usage experiences exerting a pivotal role in shaping them. Students are more likely to perceive the value of AI intuitively when they have effective and seamless experiences, and such positive perceptions can translate into greater acceptance and more favorable evaluations of AI (Niloy et al., 2024; Ntoa, 2025). Therefore, this study proposes the following hypothesis:

**H3.**  *University students’ in-class AI use is positively associated with their attitudes toward in-class AI use.*

AI can reduce cognitive load by automating tasks and providing instant answers, enabling faster and less effortful task completion and allowing learners to perceive improvements in their efficiency and productivity (Noy & Zhang, 2023; Sana & Dissanayake, 2025). Such improvements in efficiency contribute to perceived usefulness, a core positive determinant of attitudes in the Technology Acceptance Model (TAM) (Davis, 1989; Davis & Venkatesh, 1996). Cognitive relief derived from AI use may also directly improve the emotional states of users (Becker et al., 2025) and significantly reduce their anxiety (Haider et al., 2024), and when AI use makes learners feel more relaxed and less stressed, their attitudes toward the tool are likely to become more positive. Additionally, AI can support students in comprehending challenging subjects and completing demanding tasks by breaking down information and providing scaffolding (Becker et al., 2025; Song, 2025; Wang et al., 2025), which may enhance their sense of competence. This empowering experience can strengthen users’ reliance on and favorable impressions of the tool. Based on the above, this study proposes the following hypothesis:

**H4.**  *Cognitive relief positively mediates the relationship between in-class AI use and attitudes toward in-class AI use.*

Cognitive offloading may be associated with a decline in critical thinking and decision-making abilities (Gerlich, 2025b; Zhai et al., 2024), potentially causing students to notice a deterioration in their thinking capacity and foster more cautious or negative attitudes toward AI. Additionally, it may also contribute to superficial learning and impaired long-term retention (Khalil et al., 2025; Kosmyna et al., 2025), as well as reduced creativity and problem-solving abilities (Alhur et al., 2025; Haider et al., 2024). Furthermore, excessive reliance on AI may hinder the formation of deeper mental schemas (Oakley et al., 2025) and be perceived as a reflection of deteriorating academic skills (Miranda et al., 2025). These experiences can induce anxiety and worry in students (Dergaa et al., 2024), contributing to more negative evaluations of AI use. Moreover, cognitive offloading may reduce students’ sense of agency in the learning process, leading them to perceive a shift in themselves from active creators to passive editors, which can undermine intrinsic satisfaction (Zhai et al., 2024) and reduce positive attitudes toward AI use. Based on the above, this study proposes the following hypothesis:

**H5.**  *Cognitive offloading negatively mediates the relationship between in-class AI use and attitudes toward in-class AI use.*

## 3. Methods

### 3.1. Survey Sample

The data used in this study were derived from a larger survey project that covers all variables examined in the present research. All participants were recruited from a single institution, H University (a research university in China). An online questionnaire was developed using the Wenjuanxing platform, a major domestic survey tool. Convenience sampling was adopted. Members of the research team distributed the questionnaire link via WeChat, a widely used social media platform in China, to various types of student groups within H University. These groups included class groups, course groups, student club/organization groups, and major-based departmental groups. To encourage response rates, small monetary incentives in the form of digital “red packets” (hongbao) were sent together with the questionnaire link in each WeChat group. Before accessing the survey questions, all potential participants were presented with a study description, an assurance of anonymity, and a statement that participation was entirely voluntary. Participants were required to confirm their willingness to participate before proceeding. They could close the questionnaire page at any time during the process to withdraw without providing a reason, and this would not have any adverse effect on them. All responses were collected anonymously.

The survey targeted undergraduate and postgraduate students from various schools and departments at H University. All respondents participated voluntarily. Among the 287 participants, 169 were male and 118 were female; 180 were undergraduates and 107 were postgraduates; 202 had nature science and engineering backgrounds and 85 had humanities and social sciences backgrounds.

### 3.2. Instruments

Engagement was selected as the primary analytical criterion for the following interrelated reasons: Engagement captures the dynamic and sustained nature of user interaction with AI tools, reflecting not merely whether AI is adopted but also how intensively, frequently, and attentively it is integrated into learning activities (Fredricks et al., 2004). In the context of the cognitive relief–offloading paradox, engagement provides a diagnostic lens: high engagement accompanied by critical reflection may indicate the productive use of AI for cognitive relief, whereas high engagement characterized by the automatic acceptance of AI outputs may signal problematic cognitive offloading. Distinguishing engagement from the construct of digital readiness is important. Readiness refers to the preconditions for access to AI technologies, while engagement reflects the quality of actual usage. This study focuses on engagement precisely because it is more proximal to learning outcomes and more sensitive to the conflicting tensions between cognitive relief and cognitive offloading inherent in real-world AI use. The scale items for the four variables were not directly borrowed from scales adopted in previous studies but were newly developed for this research with reference to the existing literature. Accordingly, rigorous reliability and validity assessments were performed on these scales.

The in-class AI use behavior scale comprises five items and was designed based on practical experience and the relevant literature (Guo et al., 2025; Otermans et al., 2025). The scale employs a five-point Likert scale (1 = Never; 2 = Occasionally; 3 = Moderately often; 4 = Frequently; 5 = Very frequently).

The scale measuring perceived cognitive relief consists of four items and was designed with reference to prior studies (Hwang et al., 2013; Wang et al., 2025). Similarly, the perceived cognitive offloading scale comprises four items and was developed based on the related literature (Gerlich, 2025a; Miranda et al., 2025). The scale measuring students’ attitudes toward in-class AI use consists of three items and was developed by the authors. Prior to data collection, the three-item attitude scale was reviewed by three researchers to establish content validity. All three scales adopt a five-point Likert scale (1 = Strongly Disagree; 5 = Strongly Agree).

All items were finalized after adjustments based on feedback from three researchers to ensure their suitability for the in-class AI use context (See Appendix A for the specific items of each scale):

In-Class AI Usage (CU): This variable measures the frequency with which students use AI to support learning in classroom settings. Higher scores indicate more frequent use across learning activities such as answering teachers’ questions, completing quizzes, performing design or analytical tasks, and assisting with group discussions and writing.

Perception of Cognitive Relief (CR): This variable captures students’ perceptions of reduced cognitive burden resulting from in-class AI use. Higher scores indicate stronger agreement that in-class AI use helps alleviate cognitive burden by facilitating understanding of difficult concepts, assisting in task completion, helping solve complex problems, and enabling better alignment with the instructional pace of teachers.

Perception of Cognitive Offloading (CO): This variable measures students’ perceptions of cognitive offloading associated with in-class AI use. Higher scores represent stronger student recognition that in-class AI use may reduce deep thinking, promote superficial learning, increase reliance on AI-generated solutions, and discourage independent exploration.

Attitude toward In-Class AI Use (AT): This variable assesses students’ attitudes toward in-class AI use. Higher scores reflect more positive attitudes, including the approval of appropriate AI use and the recognition of AI’s role in improving learning quality and efficiency.

Control Variables: Demographic characteristics, such as gender, grade, and major field of study, were used as control variables.

### 3.3. Data Analysis

The dataset analyzed in this study is available in the Supplementary Materials. Data were analyzed using SPSS 25.0 and AMOS 25.0. The analyses included reliability and validity testing, descriptive statistics, structural equation modeling (SEM) for hypothesis testing, and bootstrap procedures to examine mediating effects.

Because all variables were assessed using a single self-report survey administered at one time point, there was a potential risk of common method bias. Harman’s single-factor test was conducted to address this concern. Specifically, all measurement items were entered into an unrotated principal component factor analysis. The results show that the first factor accounted for 35.47% of the total variance, which is below the recommended threshold of 40%. Thus, common method bias is unlikely to be a serious issue in this study. However, it is important to note that Harman’s single-factor test is not the most precise method for detecting common method variance.

## 4. Results

### 4.1. Scale Reliability

Cronbach’s alpha was adopted to test the reliability of the scales (see Table 1). All four constructs exceeded the 0.80 threshold, indicating good reliability.

### 4.2. Scale Validity

The questionnaire developed in this study was based on the existing literature and was revised based on feedback from three researchers, supporting its content validity. Confirmatory Factor Analysis (CFA) was conducted using AMOS 25.0 software to assess the measurement model. The results (see Table 2) indicated excellent model fit (χ^2^/df = 2.260; CFI = 0.942; TLI = 0.929; RMSEA = 0.075; and SRMR = 0.058), with all indices meeting the recommended thresholds (χ^2^/df < 3; CFI > 0.90; TLI > 0.90; RMSEA < 0.08; and SRMR < 0.08) (Hair et al., 2010; Kline, 2023).

The internal quality of the measurement model was assessed (see Table 3). All items loaded significantly onto their respective latent constructs, with standardized factor loadings ranging from 0.696 to 0.886 (*p* < 0.001). Composite reliability (CR) values ranged from 0.845 to 0.901, exceeding the 0.70 benchmark. The average variance extracted (AVE) values ranged from 0.521 and 0.723, all above the 0.50 criterion, confirming adequate convergent validity (Fornell & Larcker, 1981; Hair et al., 2010; Nunnally & Bernstein, 1994).

Discriminant validity was also assessed, and the square root of each construct’s AVE was greater than its correlations with other constructs, indicating acceptable discriminant validity (see Table 4). Discriminant validity was further tested using the HTMT criterion. All HTMT values were below 0.85, indicating good discriminant validity among all constructs (Henseler et al., 2015). Specifically, the HTMT value between CR and AT was 0.748, which is well below the stringent threshold of 0.85, further confirming that CR and AT are two distinct constructs in this study.

### 4.3. Descriptive Statistics and Correlation Analysis

Table 5 presents the correlation matrix, means, and standard deviations for all study constructs. The mean values ranged from 2.94 to 4.16. The correlation coefficient between CR and CO was not statistically significant (r = −0.094).

### 4.4. Hypothesis Testing

**Figure 2:**
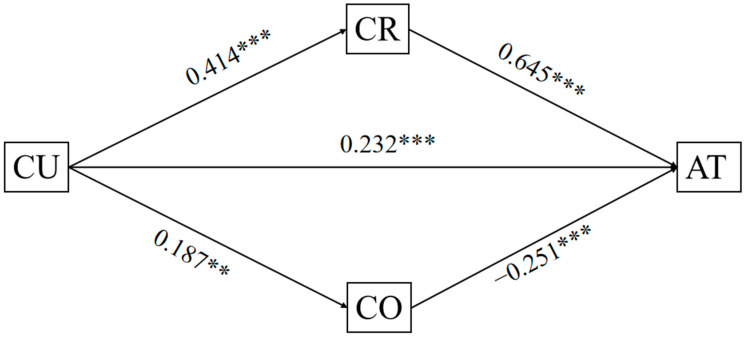
Mediation path diagram. Note: ** *p* < 0.01; *** *p* < 0.001. CU: in-class AI usage; CR: perception of cognitive relief; CO: perception of cognitive offloading; AT: attitude towards in-class AI use.

SEM was conducted via AMOS 25.0 to test the hypothesized relationships among CU, CR, CO, and AT. The path coefficients are presented in Figure 2, the SEM results are summarized in Table 6, and the mediating effects are shown in Table 7.

As shown in Table 6, CU had a significant positive effect on CR (β = 0.414, *p* < 0.001), supporting H1. CU also positively influenced CO (β = 0.187, *p* < 0.01), supporting H2. In turn, CR showed a strong positive effect on AT (β = 0.645, *p* < 0.001), while CO exerted a significant negative effect on AT (β = −0.251, *p* < 0.001). Finally, the direct path from CU to AT was significant and positive (β = 0.232, *p* < 0.001), supporting H3.

Bootstrapping results (Table 7) with 5000 resamples revealed that both CR and CO significantly mediated the relationship between CU and AT. The total effect of CU on AT was significant (β = 0.452, 95% CI [0.302, 0.580]), comprising a direct effect (β = 0.232, 95% CI [0.109, 0.352]) and two indirect effects.

The indirect path via CR (CU→CR→AT) was positive and significant (β = 0.267, 95% CI [0.170, 0.386]), indicating complementary mediation (Hair et al., 2021) through CR, supporting H4. Conversely, the indirect path via CO (CU→CO→AT) was negative and significant (β = −0.047, 95% CI [−0.109, −0.008]), confirming competitive mediation (Hair et al., 2021) and supporting H5.

Overall, the effect of CU on AT is partially mediated by both CR (enhancing attitudes) and CO (suppressing attitudes).

A bias-corrected bootstrap method (5000 resamples) was applied to formally test the significance of the difference between the two indirect effects, which was 0.314, with a 95% confidence interval of [0.155, 0.345]. This interval does not contain zero, indicating that the mediation effect of CR is significantly larger than that of CO, supporting the asymmetric effect.

**Figure 3:**
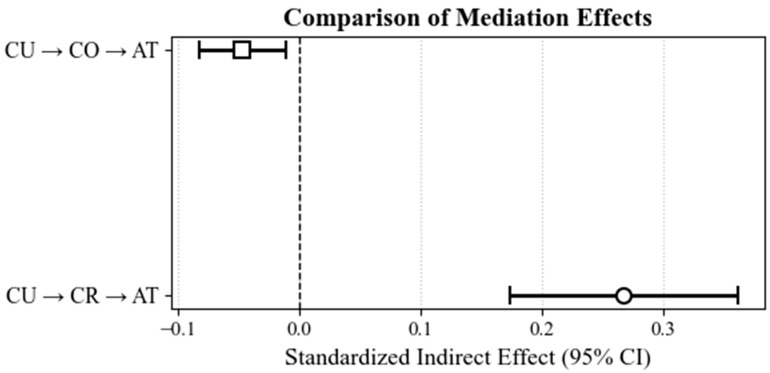
Diagram comparing mediation effects.

This study used standardized indirect effect sizes to construct a comparative mediation effects diagram to visually present the relative magnitude and significance of the two mediating pathways (see Figure 3).

## 5. Discussion

### 5.1. Theoretical Implications

Existing studies have extensively reviewed the dual nature of AI use (Chirayath et al., 2025; Jose et al., 2025a; Khalil et al., 2025; Shalu et al., 2025). However, in-depth investigations into how this duality shapes the attitudes of users remain scarce. In response to this gap, this study develops a research model centered on the dialectic between cognitive relief and cognitive offloading, explaining the impact of conflicting perceptions on attitude formation. The existing research has primarily examined variables with contradictory effects in isolation (Gerlich, 2025b; Wang et al., 2025), leaving a lack of empirical insight into how such experiences interact. This study addresses this limitation by empirically validating a dual-path model of attitude formation and verifying the contradictory relationship between cognitive relief and cognitive offloading.

Dual patterns of association linked to AI use. The correlational findings reveal pattern-based evidence for two distinct cognitive pathways that are theoretically expected to link in-class AI use with the attitudes of students. Specifically, the observed associations are consistent with a complementary role for perceived cognitive relief, which shows positive correlations with both AI use and favorable attitudes. In contrast, the pattern for cognitive offloading is consistent with a competitive process, as it is positively correlated with AI use but negatively correlated with favorable attitudes, a configuration that aligns with the notion of undesirable cognitive dependency. Together, these dual patterns suggest that AI use may be associated with simultaneous and opposing psychological forces rather than a unidimensional influence.

Asymmetry in the correlational patterns of cognitive relief and cognitive offloading. Although perceived cognitive relief and cognitive offloading show opposing associations with student attitudes, the magnitudes of these associations differ substantially. The positive association between cognitive relief and attitudes is significantly stronger than the negative association between cognitive offloading and attitudes. Specifically, the mediating effect of cognitive relief (CU→CR→AT) yielded a standardized estimate of 0.267 (95% CI [0.170, 0.386]), whereas the mediating effect of cognitive offloading (CU→CO→AT) was only −0.047 (95% CI [−0.109, −0.008]). This asymmetry suggests that positive cognitive experiences may be more closely linked to attitudes than negative dependency-related perceptions. Thus, the attitudes of students are more strongly correlated with what AI relieves than with what it offloads. These findings provide insights into how conflicting psychological perceptions are associated with attitudes toward AI use.

This study found a competing mediation effect between cognitive relief and cognitive offloading. Specifically, the same in-class AI application can simultaneously trigger two opposite psychological processes. On the one hand, AI reduces students’ immediate subjective cognitive load, generating a sense of cognitive relief (Becker et al., 2025), which, in turn, fosters more positive attitudes toward AI. On the other hand, AI may also lead students to delegate cognitive tasks externally without sufficient personal engagement (Dergaa et al., 2024). This process, known as cognitive offloading, triggers the metacognitive awareness of reduced personal investment, thereby resulting in less favorable attitudes toward AI. These two processes operate in parallel but in opposite directions. Drawing on dual-process theory (Kahneman, 2011; Milli et al., 2021), cognitive relief operates through the fast, affective, experiential system (System 1), while cognitive offloading implicates the metacognitive monitoring of one’s own cognitive effort (System 2). Therefore, the net effect of AI use on the attitudes of students depends on which pathway dominates. When the cognitive relief pathway is stronger, students are likely to develop positive attitudes; when the cognitive offloading pathway prevails, negative attitudes may emerge. This theoretical insight helps explain the mixed findings in the existing literature, where some studies report positive attitudes toward AI in education (Mazaheriyan & Nourbakhsh, 2025) while others highlight concerns about over-reliance, cognitive passivity, and even cognitive overload (Tian & Zhang, 2025). By distinguishing between these two concurrent mechanisms, our study offers a more nuanced understanding of how AI shapes the psychological responses of students in learning contexts.

### 5.2. Practical Implications

A balanced understanding of the tensions in AI use. The emergence of new technologies is often accompanied by cognitive conflict and conceptual divisions (Cecutti et al., 2021; Sparrow et al., 2011), which can trigger confusion and contradictions. Educators play a key role in helping students develop a solid understanding of the dual impacts of AI use. Alongside highlighting its conveniences and challenges, educators need to address the psychological burdens that may arise from these tensions, such as anxiety, confusion, or guilt (Chan, 2025; Qu & Wang, 2025; Zhu et al., 2024; Carter et al., 2025). Doing so can support students in maintaining psychological balance and fostering developmental resilience amid changes (Chirayath et al., 2025; Yang & Yang, 2025). AI-induced stress brings considerable psychological costs beyond immediate task performance, and these costs are linked to digital well-being, a concept that emphasizes positive mental states rather than just freedom from harm in technological settings (Song, 2025). Future research could treat digital well-being as an outcome of AI use and examine its role in mitigating the negative impacts of cognitive offloading.

The role of cognitive relief in supporting higher-order learning. The survey results show that students reported higher levels of perceived cognitive relief, suggesting that AI use is primarily experienced as beneficial in reducing cognitive load and helping to overcome difficulties and obstacles in learning. Students should be encouraged to use AI rationally in classrooms and to leverage its positive role in cognitive relief by delegating tasks unrelated to core learning objectives to it, thereby enabling cognitive resources to focus on higher-order activities, such as creative, strategic, and complex tasks (Becker et al., 2025; Chirayath et al., 2025; Miranda et al., 2025; Zhai et al., 2024).

The risks of cognitive offloading and their mitigation. The survey results indicate that students reported moderate levels of cognitive offloading, reflecting a cautious mindset. Students perceive that AI use is associated with cognitive offloading, which, in turn, is negatively associated with their attitudes toward AI use. It is recommended that educators not overlook the potential cognitive risks associated with AI use. Instead, educators should foster critical thinking and reflective learning to guard against the potential erosion of cognitive development caused by over-reliance (Khalil et al., 2025; Jose et al., 2025a).

Building on the finding that cognitive relief and cognitive offloading operate as two opposing mechanisms, educators can design interventions that maximize the former while minimizing the latter, such as the following: (1) Educators can design AI tasks that require active cognitive engagement and not allow students to directly copy AI-generated answers. Instead, they should design assignments where AI serves as a scaffold (e.g., providing hints, generating counterarguments, or offering multiple perspectives) while requiring students to evaluate, synthesize, or critique the AI output. This approach reduces passive cognitive offloading while preserving the benefits of cognitive relief. (2) Educators should teach metacognitive monitoring skills, and institutions should incorporate short training modules on how to use AI mindfully. Moreover, students should learn to recognize when they are offloading thinking unnecessarily (e.g., accepting AI answers without checking) and develop habits of self-questioning (e.g., “Do I understand why the AI gave this answer?”). Structured metacognitive prompts can help students engage with AI as a thinking partner rather than as a thinking substitute. (3) Educators can structure AI usage phases and introduce a “think-first, then-AI” rule. For example, students first attempt a task on their own and then consult AI for feedback or expansion. This sequence preserves cognitive relief while minimizing offloading-induced passivity. Additionally, time-bound AI use (e.g., allowing AI assistance during brainstorming but not during final synthesis) may prevent habitual offloading. (4) Educators can foster group discussion around AI output by having students compare their own answers with AI-generated ones in small groups, discussing discrepancies and justifications, which would convert individual passive offloading into socially active learning.

### 5.3. Research Limitations and Future Directions

First, the generalizability of our findings is limited by the characteristics of our sample. Data were collected from students at a single university in China using convenience sampling, and the sample was disproportionately male and skewed toward STEM majors, which is a significant limitation because attitudes toward AI are likely shaped by cultural, educational, and disciplinary contexts. For example, STEM students may differ from humanities or social science students in their familiarity with and trust in AI technologies (Malmström et al., 2024). Future research should prioritize diverse, representative sampling to test the cross-cultural and -disciplinary robustness of the observed patterns.

Second, this study relied on self-reported questionnaire data, which may have introduced bias. In particular, students may have underestimated their level of cognitive offloading due to their lack of awareness of the phenomenon, thereby affecting the observed mediating effects. Future research could incorporate qualitative methods such as interviews and classroom observations to explore the mediating roles of cognitive relief and cognitive offloading in the relationship between AI use behavior and attitudes.

Thirdly, this study uses a cross-sectional design, which limits all interpretations to correlational claims. Reverse causality (e.g., attitudes predicting AI use), reciprocal relationships, and unmeasured third-variable confounds cannot be ruled out. Causal mediation cannot be tested with cross-sectional data; thus, the proposed mediating roles of cognitive relief and cognitive offloading remain tentative. Longitudinal or experimental designs are needed to establish directionality.

Finally, future research could extend the research model by examining additional pairs of contradictory variables affecting attitudes toward AI use to make the study more convincing. Variables such as AI literacy, AI trust, educational level, professional expertise, structured prompting, and academic pressure have been shown to significantly impact cognitive relief and cognitive offloading (Gerlich, 2025a; Zhang et al., 2025; Wahn & Schmitz, 2024; Miranda et al., 2025) and may therefore be integrated into future research models.

In addition, while the present study focuses on Chinese higher education students, cross-national comparative research would help contextualize these findings. Different educational systems vary in their class sizes, assessment formats, degrees of digital infrastructure integration, and emphases on memorization versus critical thinking—all of which may moderate the relationship between AI use, cognitive relief, and offloading. Future studies should employ cross-national datasets to test the generalizability of the dual-path model and identify cultural or institutional factors that amplify or attenuate each mechanism.

## 6. Conclusions

Growing attention has been paid to the dual nature of AI use. However, the contradictory effects can create challenges for users in both ethical evaluation and practical decision making. This study proposes a model of attitude formation toward AI use that incorporates the contradictory constructs of cognitive relief and cognitive offloading, which jointly influence attitudes through their opposing, interactive effects. The results of this study indicate that perceived cognitive relief has positive associations with both AI use behavior and attitudes, consistent with a complementary mechanism. In contrast, cognitive offloading is positively associated with AI use behavior but negatively associated with attitudes, a pattern consistent with a competitive mechanism. Moreover, the positive association between cognitive relief and attitudes is significantly stronger than the negative association between cognitive offloading and attitudes. This asymmetry is consistent with the observation that students reported generally positive attitudes toward the use of AI. The findings highlight the importance of helping students understand the dual nature of AI use. Educators should support students in managing the confusion and psychological anxiety caused by conflicting value judgments.

Based on the empirical findings of this study, we offer several recommendations. Institutions should consider integrating specific modules focused on enhancing AI literacy and critical reflection into their curricula. These modules need not be standalone courses; rather, they can be embedded within existing disciplinary contexts. Moreover, educators should design collaborative activities that help learners complete assignments with AI support rather than simply assign AI-mediated tasks as independent work. Collaborative formats, such as small-group discussions and comparing AI-generated answers, or peer reviews of AI-assisted submissions, can foster active engagement and reduce the risk of passive offloading. AI tools themselves should be designed with pedagogical guardrails, such as requiring users to articulate their own reasoning before revealing AI suggestions, or prompting metacognitive questions during interaction. These design features can help preserve cognitive engagement while still offering the benefits of cognitive relief.

This study makes contributions to both theory and practice. Theoretically, it advances the literature by empirically validating a dual-path model of attitude formation, establishing the contradictory relationship between cognitive relief and cognitive offloading as a core mechanism shaping the attitudes of users toward AI in educational contexts. In doing so, it moves beyond the binary perspective of AI as either beneficial or harmful, offering a more nuanced framework for understanding how the same technology can simultaneously produce opposing psychological effects. Practically, the findings provide guidance for educators, instructional designers, and policymakers. Specifically, the results suggest that effective AI integration should focus not only on maximizing efficiency and cognitive relief but also on actively mitigating the risks of cognitive offloading.

## Figures and Tables

**Table 1:** Cronbach’s alpha coefficients of scales.

Variable	CU	CR	CO	AT
Number of Items	5	4	4	3
Cronbach’s α	0.843	0.898	0.877	0.884

**Table 2:** Measurement model fit.

Fit Indices	χ^2^/df	CFI	TLI	RMSEA	SRMR
Model Results	2.600	0.942	0.929	0.075	0.058

**Table 3:** Results of CFA.

Construct	Item	Standardized Factor Loading	AVE	CR
CU	CU1	0.724	0.521	0.845
	CU2	0.729		
	CU3	0.736		
	CU4	0.724		
	CU5	0.696		
CR	CR1	0.878	0.695	0.901
	CR2	0.808		
	CR3	0.860		
	CR4	0.784		
CO	CO1	0.736	0.642	0.877
	CO2	0.753		
	CO3	0.821		
	CO4	0.886		
AT	AT1	0.834	0.723	0.887
	AT2	0.843		
	AT3	0.874		

**Table 4:** Discriminant validities for measurement model.

Construct	CU	CR	CO	AT
CU	**0.722**			
CR	0.372	**0.834**		
CO	0.136	−0.094	**0.802**	
AT	0.387	0.669	−0.244	**0.850**

Note: The bold numbers on the diagonal represent the square root of the AVE for each construct.

**Table 5:** Means, standard deviations, and correlations among study variables.

Construct	CU	CR	CO	AT
CU	1			
CR	0.372 **	1		
CO	0.136 *	−0.094	1	
AT	0.387 **	0.669 **	−0.244 **	1
Mean	3.56	4.09	2.94	4.16
S.D.	0.828	0.655	0.890	0.630

Note: ** *p* < 0.01; * *p* < 0.05.

**Table 6:** SEM results.

Path	Standardized Estimate	Estimate	S.E.	C.R.	*p*
CU→CR	0.414	0.334	0.056	5.974	***
CU→CO	0.187	0.224	0.082	2.741	**
CR→AT	0.645	0.576	0.055	10.432	***
CO→AT	−0.251	−0.151	0.030	−5.001	***
CU→AT	0.232	0.167	0.042	3.939	***

Note: ** *p* < 0.01; *** *p* < 0.001.

**Table 7:** Test of mediating effects.

Standardized Effects	Estimate	Bias-Corrected 95% CI	
		Lower	Upper
Standardized total effects			
CU→AT	0.452	0.302	0.580
Standardized direct effects			
CU→AT	0.232	0.109	0.352
Standardized indirect effects			
CU→CR→AT	0.267	0.170	0.386
CU→CO→AT	−0.047	−0.109	−0.008

## Data Availability

Data is contained within the Supplementary Materials. Further inquiries can be directed to the corresponding authors.

## Supplementary Materials

The following supporting information can be downloaded at: https://www.mdpi.com/article/10.3390/bs16061014/s1.

## Abbreviations

The following abbreviations are used in this manuscript:

CU	In-Class AI Usage
CR	Perception of Cognitive Relief
CO	Perception of Cognitive Offloading
AT	Attitude towards In-Class AI Usage

## Appendix A

**Items of the In-Class AI Usage (CU)**
  Using AI to help answer questions posed by the instructor during class.
  Using AI to help complete in-class quizzes or exercises.
  Using AI to assist in completing in-class tasks (e.g., Scheme Planning, project design, case analysis).
  Using AI to support in-class group discussions.
  Using AI to assist with writing-related tasks during class.
**Items of the Perception of Cognitive Relief (CR)**
  Using AI in class helps clarify difficult concepts, making learning easier.
  Using AI in class assists in completing tasks, reducing academic pressure.
  Using AI in class aids in solving challenging problems, making learning more efficient.
  Using AI in class helps me better keep pace with the teacher’s instruction.
**Items of the Perception of Cognitive Offloading (CO)**
  Using AI in the class hinders my ability to engage in deep thinking about the learning material.
  Using AI in the class results in my acquisition of only superficial knowledge.
  Using AI in the class leads me to consistently rely on AI-generated solutions.
  Using AI in the class reduces my willingness to think and explore independently.
**Items of the Attitude towards the In-Class AI Usage (AT)**
  I support the appropriate use of AI tools by students during class.
  The use of AI during class helps improve students’ learning quality.
  The use of AI during class helps improve students’ learning efficiency.
